# “Neuropsychiatric manifestation of hyponatremia: a case report”

**DOI:** 10.1192/j.eurpsy.2024.1332

**Published:** 2024-08-27

**Authors:** N. El Moussaoui, S. Bahetta, L. Tbatou, S. Belbachir, A. Ouanass

**Affiliations:** Arrazi University Psychiatric Hospital, Salé, Morocco

## Abstract

**Introduction:**

“Electrolyte abnormalities are commonly encountered in daily clinical practice, and their diagnosis relies on routine laboratory results. Electrolyte disturbances can affect the brain among many other organs and tissues and must be promptly recognized, as they can lead to serious and potentially life-threatening complications if neglected or not appropriately treated. Neurological manifestations reflect the severity of acute neuronal dysfunction and thus require urgent treatment. Acute and/or severe electrolyte imbalances can manifest with rapidly progressive neurological symptoms, seizures, and psychiatric manifestations. They are more frequently observed in patients with sodium disorders (especially hyponatremia), hypocalcemia, and hypomagnesemia.

**Objectives:**

Were the psychiatric manifestations secondary to hyponatremia or epilepsy? Or is it a comorbidity? What are the risk factors? And what is the appropriate course of action for this type of patient?”

**Methods:**

We present, through a clinical case, the situation of a 64-year-old patient who experienced status epilepticus secondary to hyponatremia, requiring hospitalization in the neurology department. Subsequently, she developed psychiatric manifestations with a marked change in behavior. She began experiencing symptoms of anxiety and depressive mood, headaches, somatic complaints, and social isolation. Her condition gradually worsened, necessitating hospitalization in the psychiatry department 3 years later.

**Results:**

The patient was placed on Carbamazepine by her neurologist, and since then, she has not experienced epileptic seizures. Her follow-up electrolyte panel initially showed slight disturbances before normalizing. Psychiatric manifestations were concurrent with these somatic symptoms and worsened over time. During her psychiatric hospitalization three years later, after a thorough evaluation, she was prescribed Sertraline ans Risperidone in combination with Carbamazepine, resulting in a significant improvement in her condition.

**Conclusions:**

In summary, this case illustrates the critical impact of electrolyte abnormalities on both neurological and psychiatric health, especially in older patients. Understanding risk factors associated with electrolyte imbalances is crucial for effective diagnosis and management, particularly in the elderly. This underscores the importance of a multidisciplinary approach to address the potential serious consequences of electrolyte disturbances on overall patient well-being.

**Disclosure of Interest:**

None Declared

